# Pooling the ground: understanding and coordination in collective sense making

**DOI:** 10.3389/fpsyg.2014.01233

**Published:** 2014-11-07

**Authors:** Joanna Rączaszek-Leonardi, Agnieszka Dębska, Adam Sochanowicz

**Affiliations:** ^1^Psycholinguistics and Cognitive Psychology Lab, Institute of Psychology, Polish Academy of SciencesWarsaw, Poland; ^2^Psycholinguistics Lab, Faculty of Psychology, University of WarsawWarsaw, Poland

**Keywords:** dialog, common ground, perspective-taking, coordination dynamics, language

## Abstract

Common ground is most often understood as the sum of mutually known beliefs, knowledge, and suppositions among the participants in a conversation. It explains why participants do not mention things that should be obvious to both. In some accounts of communication, reaching a mutual understanding, i.e., broadening the common ground, is posed as the ultimate goal of linguistic interactions. Yet, congruent with the more pragmatic views of linguistic behavior, in which language is treated as social coordination, understanding each other is not the purpose (or not the sole purpose) of linguistic interactions. This purpose is seen as at least twofold (e.g., Fusaroli et al., 2014): to maintain the systemic character of a conversing dyad and to organize it into a functional synergy in the face of tasks posed for a dyadic system as a whole. It seems that the notion of common ground is not sufficient to address the latter character of interaction. In situated communication, in which meaning is created in a distributed way in the very process of interaction, both common (sameness) and privileged (diversity) information must be pooled task-dependently across participants. In this paper, we analyze the definitions of common and privileged ground and propose a conceptual extension that may facilitate a theoretical account of agents that coordinate via linguistic communication. To illustrate the usefulness of this augmented framework, we apply it to one of the recurrent issues in psycholinguistic research, namely the problem of perspective-taking in dialog, and draw conclusions for the broader problem of audience design.

## INTRODUCTION

In most traditional approaches to language, sense-making happens at the individual level. Language itself is seen as an information carrier, in which vessels for meaning arrive from the speaker, and are unpacked by the addressee by means of complex computational processes over pre-existing representations (e.g., Katz, 1966; Frazier and Clifton, 1996). Even when the dialogical nature of linguistic communication in guiding production and comprehension is acknowledged, as in recent mechanistic models (Pickering and Garrod, 2004, 2013), the process of communication has a similar, information-transmitting character: it’s goal is most often for participants to understand each other, which consists of making their situation models as similar as possible.

Recently, however, an increasing number of approaches have investigated language in a more ecological setting of situated social coordination. The approaches vary from more pragmatic ones, which regard language as a tool for social coordination, to more radical ones, in which it is the linguistic interaction itself that temporarily transforms individual cognition and constitutes social coordination. Neither of these approaches considers understanding each other to be the ultimate goal of an interaction. Rather, the aim is to form (or to become) a temporary functional system, jointly structured by environmental requirements^1^.

In a recently proposed model of dialog as interpersonal synergy (Fusaroli et al., 2014), this systemic and functional character of linguistic interaction is given a more systematic form. This model is based on an assumption that language, instead being a system of meaning carriers is rather a system of constraints on an ongoing, situated interaction. Due to the history within a culture and within development in this culture, language has the power to functionally control^2^ the interaction as a whole (Rączaszek-Leonardi and Scott Kelso, 2008; Rączaszek-Leonardi and Cowley, 2012). Such a perspective on language, in which interaction in a concrete situation is constitutive of the meaning of utterances, brings several major changes to the way explanations of linguistic behaviors are constructed:

• First, global characteristics that pertain to the level of a conversing dyad become a valid source of explanatory variables, on par with individual cognitive processes. Global and local processes are in a relationship of co-construction, or circular (reciprocal) causality (e.g., Kelso, 1995). Thus, equally important as the study of individual processes, it is important to study features of global organization such as task-functionality, stability, dimensionality, reaction to perturbation, etc. These global features can be related to local, individual processes (behaviors and experiences), which opens ways of understanding and modeling both the emergence of global characteristics from the local ones and the transformative effects of interaction on individual cognitive processes.
• Second, a system created in a conversation is qualitatively new. The meaning created in a distributed and participatory way can be neither described nor predicted by the analysis of conceptual or linguistic knowledge of the participants individually (e.g., De Jaegher and Di Paolo, 2007). It arises in a dynamical, dialogical relationship between the participants under the constraints of a situation. The system’s existence and the states it assumes actualize one of many possible ways to organize a dyad, in direct contact with environmental constraints.
• Third, congruent with the general notion of synergy, the model proposes that two distinct mechanisms are necessary for creating a functional system in dialog. On the one hand, there are mechanisms responsible for making the system coherent and sustainable; on the other hand, there are mechanisms for distributing the roles among the elements of the system to effectuate a functional coordination (Fusaroli et al., 2014). This twofold character is manifested both at the physical level of interaction and on the semantic (or content) level^3^.

Recent research has uncovered a variety of mechanisms for maintaining physical coherence in interacting individuals, such as similarity in time (synchrony) and space (imitation). Some studies have also investigated mechanisms for physical complementarity, which involved reciprocity of movement (van Schie et al., 2008; Sartori et al., 2011) or turn-taking structure (Wilson and Wilson, 2006). Yet the search for mechanisms that provide coherence and complementarity on the content level have thus far been limited to just one part of the story, namely the similarity aspect, which is, for example, achieved through priming (as in Pickering and Garrod, 2004), or, less mechanistically, through the process of grounding dialog in dynamically developed common ground (Clark, 1996). What seems to be much less developed is the conceptual apparatus, which could account for semantic complementarity, i.e., for meaningful differences that make people interact in the first place and that are integrated in a dialog, resulting in a more capable collective structure. A step in this direction is research on the emergence of dialogical scripts, in which complementary roles develop in the course of task-oriented interaction (Mills and Gregoromichelaki, 2010; Mills, 2014); however, that research pertains more to the general moves in conversation (functionally understood), while here we would like to focus on the semantic resources available for an interacting dyad.

The aims of this paper are to advocate the need for a conceptual apparatus that can encompass such semantic complementarity, to trace established concepts and approaches that can support its theoretical foundations, and to begin its construction. Realization of these aims will require the integration of the synergetic approach to dialog with more traditional dialog research, which is the main source of the key concepts. To situate language in action, we first briefly survey the ways in which the relationship between linguistic communication and coordination has been conceptualized, emphasizing pragmatic approaches that represent an ‘understanding-for-coordination’ perspective. Then, we determine which conceptual tools are already available to talk about language in coordination; namely, we analyze the notions of ‘common’ and ‘privileged’ ground and their respective role in the explanations of task-oriented linguistic encounters. Next, we propose that although the notion of dynamically accumulating, situationally relevant common ground has been indeed a step toward understanding the coordinative role of language in research on dialog, it is not sufficient to account for the distributed nature of a conversing system. For this, the notion of ‘pooled ground’ will be advanced to describe resources on which the emerging, qualitatively new, functional dialogical structure is based. Finally, we apply this augmented framework to the recurrent problems in psycholinguistics and cognitive psychology. The case we will analyze is the debate on perspective-taking in dialog. We show that what might seem like an automatic egocentric perspective (e.g., Keysar et al., 2000, 2003) may stem from the functionality of such behavior for the dyadic system as a whole. We also reflect on the applicability of the proposed notion to the broader phenomenon of audience design. The view from the level of interaction prompts to interpret audience design not only as adapting one’s speech to the listener so that she better understands it but also as designing one’s speech to seek what is missing in the speaker’s knowledge but is crucial for the joint project. Both examples will demonstrate the explanatory value of the collective level and raise questions about the proper level of analysis for linguistic structures and behaviors.

## COMMUNICATION: UNDERSTANDING AND COORDINATION

‘Understanding’ is one of the most broadly discussed concepts in both philosophy and in psychology of language; thus, reviewing, even superficially, its many facets exceeds the scope of this paper. Leaving aside the problem of understanding as grasping the meaning of a proposition in its relation to the external world, we will focus only on understanding in interaction and briefly survey the ways in which understanding is seen to relate to interpersonal coordination.

In many traditional approaches to language, understanding has been treated as a sole goal of linguistic communication. As Wittgenstein (1967, p. 114) complained: “*(…) we are so much accustomed to communication through language, in conversation, that it looks to us as if the whole point of communication lay in this: someone else grasps the sense of my words—which is something mental: he as it were takes it into his own mind. If he then does something further with it as well, that is no part of the immediate purpose of language*.” The realization of the goal of understanding each other was often described as coding and decoding a message (e.g., Katz, 1966). This made Dummet (1996, p. 97) characterize the traditional view as assuming that “c*ommunication is (…) essentially like the use of a telephone: the speaker codes his thought in a transmissible medium, which is then decoded by the hearer (…) Concepts are coded into words and thoughts, which are compounded out of concepts, into sentences, whose structure mirrors, by and large, the complexity of the thoughts*.”

The ‘code’ conception of language, or a conduit metaphor of communication (Reddy, 1979), is recently increasingly criticized both in philosophy and in psychology. It seems to fail in many ways; one of the most important is being unable to adequately address the issue of contextual flexibility (the same message could be understood to mean different things in different contexts). Without making the context (more precisely, the relevant features of the context) part of the ‘code,’ a communication model that consists simply of encoding and decoding has difficulty explaining how the same encoding can at different times yield different decodings (Barwise and Perry, 1983; Krauss and Chiu, 1997). Not of lesser importance is the fact that, as noted in Wittgenstein’s quote, the code conception of language ignores the pragmatic and performative aspects of linguistic behavior.

Accounts that attempt to embed goals of human communication in a wider social context and not restrict it only to mutual understanding have been present in the philosophical literature for quite a long time. This pragmatic aspect of linguistic communication has been emphasized, for example, in the work of Hillary Putnam, who indicated that language and linguistic behavior hold a subservient role in the global activity of the users. As he put it:

“*What succeeds or fails is not, in general, linguistic behavior by itself but total behavior. E.g. we say certain things, conduct certain reasonings with each other, manipulate materials in a certain way and finally we have a bridge that enables us to cross a river that we couldn’t cross before. And our reasoning and discussion is as much a part of the total organized behavior complex as it is our lifting of steel girders with a crane. So what I should really speak of is not the success or failure of our linguistic behavior, but rather the contribution of our linguistic behavior to the success of our total behavior* (Putnam, 1978, p. 100).”

In pragmatic approaches, the personal and contextual factors are openly admitted in the process of understanding an utterance. According to Dascal and Berenstein (2003, p. 83.), in compliance with Gricean tradition, *understanding is always pragmatic understanding. “It is not a matter only of understanding speaker’s words (determining the “sentence meaning”), but always a matter of getting to the speaker’s intention in uttering those words in that context (determining the “speaker’s meaning”).*”

Similar debates have been present in analytical philosophy, where utterance comprehension should result, according to Michael Dummet, in the recognition of interplay between conventional meaning attributed to words and sentences and the contextual determinants. The degree to which the former factors (conventionalized meaning) indeed play the role in the process of communication also varied in philosophical theories – from practically determining this process to being always modified and dependent on context. As in Davidson (1986, p. 174): “*We must give up the idea of a clearly defined shared structure which language-users acquire and then apply to cases.”* According to Davidson, what people converge on is only passing theories, and such convergence is a result of applying all possible resources at hand – both linguistic and extra-linguistic.

The tension between understanding as a goal in itself or as a means to coordination is correlated with the tension between the representative vs. performative functions of language. If the goal is just to understand each other, the representative function is emphasized and the process of communication becomes one of making these representations similar [as in the Pickering and Garrod’s (2004) model]. However, if the language’s function is sought rather in effectuating coordination, its creative and performative powers come to the forefront. We find a similar distinction in Dummet (1996, p. 185, 187), who stated that “*the true opposition is between language as representation and language as activity (…); the significance of an utterance lies in the difference that it potentially makes to what subsequently happens.”*

The debate sketched above seems to reflect, from a philosophical point of view, the controversies entailed by the relationship between understanding and overall practical coordination as a goal of communication. Both understanding and coordination rely on the similarity of knowledge between the interaction participants. However, while understanding each other seems to refer to and rely on overlapping knowledge, in practical coordination, the knowledge implicated in the deeds of the partners need not be entirely common, as long as actions are appropriate. Only if linguistic interaction is considered ‘for understanding’ can its goal be described as broadening the scope of mutually shared knowledge; when language use is seen as a control process in an ongoing interaction, leading to practical coordination, what is mutually shared is but a foundation on which something new is created in interaction. The core of the discussion can be thus seen as a question to what extent successful communication consists in broadening and strengthening a pre-existing harmony and to what extent it consists of efforts that aim to coordinate and overlap separate idiolects in the goal of creating a new quality under external constraints.

In philosophical inquiries, it has also been underlined that one’s comprehension of a given utterance can only be accessible through the manifestation of the state of understanding. Such an approach allows for a departure from considering understanding only as a private, covert, and individual process that is purely a mental phenomenon. As noted by Quine (1990, p. 58): “*In practice, we credit someone with understanding a sentence if we are not surprised by the circumstances of his uttering it or by his reaction to hearing it – provided further that his reaction is not one of visible bewilderment.*” Thus, the ‘operationalization’ of understanding (success in communication), similar to the conversational-analytic approaches, is through what happens next in the overall interaction.

We will now move to the characterization of these issues from the psycholinguistic perspective. Counterparts of the above mentioned problems in psychological and linguistic research on language involve many issues that appear when pragmatics and jointness (dialogicity) of language are addressed. In what follows, we focus on the subset of those issues, surveying the toolbox of available concepts. We begin with an overview of the notion of common ground, which constitutes a pivotal concept in addressing the above questions on both the theoretical and empirical level, and then continue to the notion of privileged ground. The sufficiency of these concepts will be evaluated for accounting for task-oriented dialog.

## COMMON AND PRIVILEGED GROUND

The notion of common ground has been most extensively used and explored in psycholinguistic theories by Clark and Marshall (1978), Clark et al. (1983), and Clark (1996). Common ground, defined as a “sum of mutual knowledge, beliefs and suppositions” (Clark, 1996, p. 93) enables agents to recognize and represent the general information about the world as well as about previous states and current situations that is shared among them. This is the basis for mutual expectations of each other’s behavior in a given stage of the task (Clark, 1996, pp. 43–49). The most important feature of common ground is thus the assumption of mutuality. It is not enough that two people have the same knowledge; they must realize that this knowledge is mutually shared.

In most psycholinguistic research, common ground has been treated as a relatively simple characterization of mutually available information, which would be a prerequisite in communication. In many experimental settings it is usually operationalized as those elements of a visual field that are accessible to both participants. Yet it is important to appreciate the complexity of this concept, its joint, dynamical, and contextualized nature. This has been most fully exposed in research on dialog, and especially in Clark’s (1996) approach, where it serves to ground conversation and to enable the principle of least collaborative effort to explain many aspects of linguistic interactions.

Clark sees a conversation as a type of rational joint action, with different levels of joint projects, hierarchically and sequentially organized (Clark, 1996; Bangerter and Clark, 2003, p. 150). A minimal joint project is understood as an adjacency pair – a proposal from Agent A to take a joint project and Agent B’s response to uptake it, like in a typical question–answer pair (Clark, 1996, pp. 191–220). Linguistic communication is a tool for the coordination of more basic actions immersed in a physical world. Some joint actions obviously do not require coordination via language, like dancing or playing a piano duet, but in most co-actions language is necessary to succeed. For example, when Ann and Bob are engaged in moving a table, they might use language for navigation in different joint projects that constitute the joint action, such as selecting a place to put it, lifting the table, lowering it together, etc. Bangerter and Clark (2003) noticed that sounds or words produced during conversation, which were traditionally considered to be turn-taking signals or emotional acts may in fact reflect the structure of the joint task, as for example, they may serve as markers that indicate the stage of the project. They analyzed corpora from experimental communication tasks and spontaneous telephone conversations (over 3.5 million words in English and German) to show that words like “okay” and “all right” served as horizontal markers (indicating the beginning and end of a particular joint project), and “m-hm,” “u-huh,” “yeah” were vertical markers (signalizing the expectation of continuation).

Although communication serves as a coordination device for joint actions, it itself needs to be coordinated. Interlocutors participate in a collaborative process (grounding) where they constantly signalize to each other their engagement in a course of events. As Clark (1996, p. 246) noticed, in the grounding process, new information is prominent when it concerns the basic level of action (in physical world or in speech act), but signals pertaining to the level of understanding “should be backgrounded.” Usually, agents involved in a joint action need to signalize if they finish or start a new project to maintain continuity and compatibility in a track of joint projects, but they tacitly assume that they accomplished the level of mutual understanding. Their assumptions might be easily violated if the other party’s behavior is not in line with expectations that emerged in the communication process (congruent with Quine’s (1990) characterization above, behavior in co-action is thus the final criterion for ascribing understanding). These expectations, though not explicitly expressed, are part of the common ground. They are built on three types of information: initial common ground (mutual knowledge that participants bring into a conversation), the current state of the joint activity, and public events that happened from the beginning of the joint action (Clark, 1996, p. 43).

Cumulative history of dialog with another person forms background information that dynamically creates a shared context. It may, for example, cause shaping utterances from long and informative to short and elliptical (Mills and Gregoromichelaki, 2010; Mills, 2011), or even result in less care in the pronunciation of words that have been mutually used in a given conversation (Fowler, 1988). The tendency to make a conversation shorter and more succinct in a shared context is consistent with the least collaborative effort principle (Clark and Wilkes-Gibbs, 1986). This principle has been evoked mostly in situations in which participants must agree on a reference, in order to explain how redundancy is kept minimal. For example, in a communication game when a director has to provide the matcher with information about the shape of tangrams (highly ambiguous, geometrical figures), his first descriptions are relatively long and detailed, but in subsequent rounds they become shorter, up to almost becoming proper names. The speaker does not have to use long utterances anymore because the dyad has developed ways to conceptualize and refer to the tangrams (Wilkes-Gibbs and Clark, 1992).

Accumulated common ground on the level of conceptualization is also responsible for the phenomenon called lexical entrainment, where a speaker refers in the same way to the same object in the interaction with the same interlocutor but might change the term in a conversation with another interlocutor (Brennan and Clark, 1996). This is also an example of applying the least collaborative effort principle (changing a referring term for the same pair without a good reason is in conflict with the conversational economy, Metzing and Brennan, 2003). Similarly, the act of perspective-taking in conversation may be useful in minimalizing the cost of future possible misunderstandings. If the interlocutors are aware that their visual perspectives differ, they will try to use terms that refer to neutral spatial descriptions (Schober, 1998).

Other aspects of common ground, understood as a shared physical, social, and linguistic environment in a current state of activity, might explain how interlocutors are able to properly interpret utterances that are strongly context-dependent, such as definite references. When Ann says to Tom, “Give me the bottle,” she most likely means the one that they both have seen or talked about previously, so Tom can safely reject interpretations that refer to his private bottle of water hidden in his bag^4^. Depending on the recognition of what is and what is not in common ground, the addressee may narrow the interpretations to those related to the speaker’s knowledge and their shared history of communication.

Thus, ‘common ground’ is a very broad construct. It focuses on everything that is recognizably shared in a conversation. Especially as construed in Clark’s theory, its incremental, dynamic, dialogical, and situated character makes it a very useful notion for explaining how people zoom in on common references or resolve ambiguities with least collective effort (e.g., Wilkes-Gibbs and Clark, 1992; Clark, 1996; Clark and Krych, 2004). Equally important, it helps determine what information would be new, i.e., what is worth volunteering in a next conversational move and worth entering into the accumulating common ground. According to Grice’s cooperative principle, the volunteered information must be based on common ground to be relevant to the course of discussion, but it must be novel enough to be a real contribution to the conversation. Saying something that actually is a part of mutual knowledge is a violation of the quantity maxim, and in usual conversation, it may turn into an implicature, such as in a situation when Bob flirts with a woman and Ann says to him: “I think you have a wife.”

However, even this dynamic, task-oriented, and joint conception of common ground does not allow to address the complementary parts of knowledge that remain private but nevertheless influence how a dyad is coordinating on the task. By focusing on what is common, mutually shared, the notion of common ground emphasizes the similarity (or coherence) aspect of the formed synergy. This, perhaps, stems from the historical provenience: the main theoretical focus of work on common ground was how people establish a common reference to external objects and much less on the distributed aspects of joint actions. The least collaborative effort principle does point to the fact that one interlocutor counts on the knowledge of the other, but it is mainly the shared knowledge. The principle was designed rather to explain curbing redundancy in speech acts than to make possible the distribution of resources, which, after all, also (if not primarily) leads to performing the tasks with least collective effort.

The synergetic approach may be useful to augment the task-immersed dialog theory with this distributive aspect by more clearly relating the ‘linguistic’ and ‘action’ projects in Clark’s approach. It proposes a specific relationship between the joint projects on the level of action and on the level of conversation. Basing on the notion of language as a constraint (Rączaszek-Leonardi and Scott Kelso, 2008; Pattee and Rączaszek-Leonardi, 2012), in this model the moves in a dialog are not viewed as containers for the transmitted content but rather as constraints on a collective project. Given the joint nature of linguistic interaction, these are jointly constructed. Thus the dynamics of both individual and joint action is regulated and guided by language rather than being expressed or described in it.

The controlling role of language in collective projects thus requires a joint establishment of task-relevant constraints using linguistic structures. This means that these two projects cannot really be understood separately: being ‘just’ a constraint, an utterance can be understood only in context of the ongoing project, as it relies for meaning on the action it constrains (Pattee and Rączaszek-Leonardi, 2012; Rączaszek-Leonardi, 2014). The two sides of a linguistic interaction: a joint pragmatic project and joint construction of constraints might nevertheless rely on different mechanisms and may provide different sources of structuring for a conversation. Thus an appearance of a given utterance at a given moment of interaction may reflect both the structure of the task and the conventionalized ways of structuring linguistic interactions so that they become effective controls in interaction.

The proposed constraining relationship between language and coordination in situated coaction leads in many cases to similar predictions as Clark’s grounding theory. For example the abovementioned shortening of expressions and increase in the use of ellipsis in the course of a conversation stem from the fact that less control is needed when more coordination is already in place. However, beyond that, accepting the constraining role of language (rather than content-conveying one) also facilitates seeing linguistic behaviors as serving a larger, distributed system. Conceptual pacts are good examples of a dyad zooming in on effective controls in a given situation; the process of emergence of the dialogical scripts can be seen in the light of their stabilizing participant’s roles in frequently recurring joint projects. The latter can take place both on the timescale of a particular interaction in a particular task (as, e.g., in Mills, 2011) and on the slower timescale, when culturally specific dialogical scripts emerge, revealing frequent structures of joint projects encountered in the social life of a particular culture (such as, for example, question–answer adjacency pairs, or “greeting chats,” which may have particular structures, cadence, and even limited contents, e.g., “weather chats” in England, or asking about the health of relatives in Poland).

Another good example of joint establishing of task-dependent effective linguistic controls comes from research on language functioning in joint decision making: e.g., Fusaroli et al. (2012) show that performance on a joint decision task depended not on unspecific lexical alignment of the participants, but rather on the dyad’s selecting-by-alignment of specific dimensions that were crucial for the task. Repetitive expressions of those dimensions, in turn, kept the actions of the participants organized around them. Importantly for the arguments presented in this paper, they would do so even if actual actions and knowledge, on which the use of those expressions was based, were idiosyncratic to each participant.

It is important to reiterate that the principle of least collaborative effort pertains to both levels of coordination: coordination of controls (where only minimally needed constraints for the ongoing interaction are jointly provided, and where partners count on each other to make constraints more precise) and the coordination of a joint project itself, when participants rely on being similar but also on each other’s idiosyncratic capabilities in the division of labor. These capabilities (skills and knowledge) might be complementary and remain unshared, as long as they do the job required for the project.

The idiosyncratic knowledge in linguistic interaction is referred to as privileged ground. While the concept of common ground has a long tradition in philosophy and psycholinguistics, the concept of privileged ground is relatively new and has been used in more limited contexts. It was construed in opposition to the common ground and is defined as knowledge that a single interlocutor attributes only to herself (for example, because she has privileged perceptual access to it; see e.g., Keysar et al., 1998). In many examples of linguistic analyses of communicative interactions and in psycholinguistic research, privileged information is usually seen as a distractor, drawing attention away from the common ground on which the interaction should stay, as dictated by the experimental tasks. If an interlocutor cannot ignore distractors present in privileged ground effectively, it is usually concluded that she shows an egocentric tendency (Keysar et al., 1998, 2000, 2003; Wardlow Lane et al., 2006; Lin et al., 2010).

Constructing tasks in this way, however, researchers limit the applicability of their results to only a subset of everyday communication situations — a subset, let us add, that is compatible with the view of ‘understanding’ as ‘equalizing world models.’ In a way, the privileged information is made irrelevant by design. Yet if the distributed character of interactions is to be taken seriously, the importance of role-division and idiosyncratic contributions to the task become evident, and, with it, the importance of the privileged ground and the ways of making the relevant elements of it bear on the task. Focusing on privileged information by an individual in interaction thus becomes a necessity, a desired thing, not an imperfection of the participant. The question, in such a distributed framework, is thus not how the common ground is broadened for understanding but how both common and relevant privileged information can be used in collaboration on a realized project^5^.

It seems that neither the notion of common ground nor privileged ground are sufficient to account for this kind of diverse but complementary influence that the participants can exert on joint projects within a distributed system. If linguistic interaction is to effectuate the coordination of a dyad toward various projects according to the least collaborative effort principle, it has to realize the division of labor: i.e., optimally using both parties’ resources, without making them common.

A similar aspect of collectivity in meaning creation through constraint construction is also visible on slower time scales and larger systems: one can recall here Hilary Putnam’s view on how the meaning of words is distributed in populations. He introduced the notion of division of linguistic labor, relating it to the performance and coordination of real-life tasks via linguistic means: *“(…) it is certainly not necessary or efficient that everyone who has occasion to buy or wear gold be able to tell with any reliability whether something is really gold. The foregoing facts are just examples of mundane division of labor (…). But they engender a division of linguistic labor: everyone to whom gold is important for any reason has to acquire the word ‘gold’; but he does not have to acquire the method of recognizing if something is or is not gold. He can rely on a subclass of speakers. (…) that collective body divides the ‘labor’ of knowing and employing these various parts of the ‘meaning’ of ‘gold’*” (Putnam, 1975, p. 141).

Returning to dyadic interactions and faster time-scales: a concept is thus needed that can account for the dyad’s ability to rely on the knowledge of both participants, however, without the condition of its mutuality. Such knowledge can be a basis for complementary behaviors in a task situation (as agents act on the basis of common and private knowledge) and for linguistic acts that may not necessarily reveal or convey information but also signalize responsibility for privileged knowledge and scout for possibly relevant information. An expression dictated by an individual’s privileged ground may thus become an active control of the dyad’s behavior, which means that information, which does not enter common ground might nevertheless be decisive for interaction. Thus the proposed concept should pertain to a dyad as a whole and should help understand the resource in which the dyad’s behavior is grounded.

## POOLING THE GROUND – A VIEW FROM INTERACTION

The view of language as a constraint on social coordination poses the creation of functional synergy, not understanding itself as the main *explanans*. The main questions thus concern how language facilitates coordination of cognition and action in concrete situations, how it controls and disambiguates possible ways of knowing and acting. In a sense, thus, it is not the context that disambiguates the word senses, as in traditional information-processing approaches, but rather utterances in a situation that actualize certain possibilities for interpretation and action and thus ‘disambiguate’ the context (Rączaszek-Leonardi and Scott Kelso, 2008; Collier, 2014). Expressions do not convey meanings but rather, once used, operate reflexively, contributing to the common context and organizing experience.

This aspect of human interaction parallels the notion of reflexivity applied by Garfinkel (1967) in his ethnomethodological studies on practical everyday activities. Garfinkel (1967, p. 8) emphasized that it is commonly “*treated as the most passing matter of fact that members’ accounts, of every sort, in all their logical modes, with all of their uses, and for every method for their assembly are constituent features of the settings they make observable.*” On this view, reflexivity means that members shape action in relation to context, while the context itself is constantly redefined through action^6^.

Thus in such ‘view from interaction,’ linguistic expressions, always immersed in co-action, effectuate dynamic changes both in individual participants (according to their history in a given culture) and on the level of a dyad, where they control interactants’ behavior in a dialogical process. Congruent with the third claim of the synergetic approach mentioned in introduction, the formation of such a functional distributed system requires both coherence of a dyad (to be a system at all) and complementarity – i.e., the division of labor, which allows for an optimal use of the resources of each participant.

The key issue for understanding language use in dialog is to identify the mechanisms, i.e., processes both on the individual and interaction level, due to which coherence and complementarity are realized^7^. In the case of physical aspects of human interactions, an increasing amount of evidence for the existence of mechanisms for maintaining coherence is described in developmental contexts where infants focus on, imitate and synchronize with adults (Meltzoff and Moore, 1977; Murray and Trevarthen, 1985; Johnson et al., 1991) and in adults (Schmidt et al., 1990; Shockley et al., 2003). Not requiring division of labor, these mechanisms function alike in different contexts, perhaps differing in strength, when, e.g., the need for social coherence is greater (for discussion on this point, see also Fusaroli et al., 2014). In linguistic interactions, one mechanism proposed for achieving similarity is priming, with its various types (semantic, syntactic, etc.).

However, mechanisms that realize the coordination of diversity in interaction, i.e., those that bring about division of labor, complementarity, flexibility, and compensation, are not as self-sufficient. They cannot be described without taking into account a specific situation of interaction. Complementarity is a complex relational concept that involves not only the cognitions and actions of participants but puts those in relation to a situation in which the interacting participants are immersed. On a physical level, mechanisms for achieving complementarity in human coaction are being uncovered. Early education of attention for co-action is visible in development (Rączaszek-Leonardi et al., 2013), as well as early signs of complementary action in anticipation to the caretaker’s movements (Reddy et al., 2013), while in adults the activation of neural structures responsible for complementary and compensatory (and not only imitative) movements have been demonstrated (van Schie et al., 2008; Sartori et al., 2011). In the language domain models are proposed for entraining antiphase in syllable rate for turn-taking (Wilson and Wilson, 2006). Yet when it comes to the content level of linguistic interaction, it seems that the mechanisms for achieving complementarity are still not worked out. As said earlier, priming and even more elaborate mechanisms for the construction of common ground, because of their focus on mutuality, will not explain the complementary aspect of this level of communication.

We propose that in forming task-dependent dyadic systems, the informational resource can be characterized as ‘pooled ground.’ This refers to the aggregate of the common ground and the relevant privileged ground that may never enter common ground (become mutual) yet is a basis for individual behavior influencing the dyad. To pool knowledge in coordinative situations, language is thus used not only to confirm a shared vision of a situation, but also to ‘scout’ for and signalize mutually *un*available resources (information or skills), which would enable efficient functioning of the global system. The necessity of the concept comes from changing perspective from the individual to the dyadic level and acknowledging its distributed nature. It does not matter if resources are shared, as long as one of the participants makes them effective in the dyad’s dialog and, eventually, behavior.

Here we use the first two tenets of the synergetic approach, mentioned in the introduction. By ascribing functionality to the entire system, we analyze individual processes as parts of this system. New variables – such as effectiveness or stability of a system as a whole – become explanatory also for the behaviors of the individual participants. The dyad, acting on the basis of unshared information, is a qualitatively new system, dependent on the interaction of the individual resources. Meaning is made in interaction due to individually produced constraints the bases of which might not be shared (i.e., the private knowledge that is the reason for their production is never expressed) but nevertheless bear on the behavior of the system.

From this perspective, the situation of communication, unlike in traditional psycholinguistic experiments, can be viewed not as relying on common ground, with elements of privileged ground distracting from perfect mutuality, but rather as relying on common ground with elements of privileged ground enabling moves (actions and utterances) that are beneficial for the overall behavior of the system yet never entering the common ground. Language thus acts as a constraint on individual and dyadic dynamics and, on the other hand, is an outcome of dynamic processes within individuals and dyads (Rączaszek-Leonardi and Scott Kelso, 2008; Pattee and Rączaszek-Leonardi, 2012).

Polanyi (1966, p. 6), in his *Tacit Dimension*, similarly describes the process of apprehending knowledge:

“*Our message had left something behind that we could not tell, and its reception must rely on it that the person addressed will discover that which we have not been able to communicate.*”

Or we may risk an even stronger claim: sometimes it is not necessary that the person make the discovery; she might rely on a partner having made it to make a next step in a joint reasoning. Communicative acts effectuate idiosyncratic changes in interlocutors, which will never be mutually available but which, in the cases of good communication, may lead to desirable collaborative outcomes.

The problem with the definition of the pooled ground lies in specifying what is enough to be known about the knowledge of the other to rely on it for the task: it is not the proposition, or any other form of a piece of factual knowledge itself, but rather consequences of acting upon it for the joint task. While the common ground requires that A know x, B know x, and they both know that they know x (Clark and Marshall, 1978; Clark, 1992, 1996), and while privileged ground means that A does not know x, B knows x and B knows that A does not know x, a task-dependent pooled ground could be described as A not knowing x, B knowing x and A knowing that B knows x^8^, which seems paradoxical without A knowing the content of x.

However the paradox dissolves if – as in the presented approach – language acts as a constraint, not as content carrier. The same expression may – to some extent –act differently on each interlocutor. For A, it might be enough to receive a signal that B knows the information needed for a task to rely on it. This is different from actually receiving this information; the content of B’s knowledge does not enter the common ground. Knowing the task constraints should help predict the use of common (mutual) ground and the use of privileged (private) ground, which could change dynamically during task-dependent interaction.

The notion of pooled ground thus goes beyond common ground. It also goes beyond “implicit common ground,” proposed by Pickering and Garrod (2004). Their conception is very helpful in finding mechanisms that establish common ground between interlocutors: it points to the possibility of its arising without inferences about, or modeling, the interlocutors’ state of knowledge. Instead, they claim, the implicit common ground arises automatically in the interlocutors by being in the same culture, situation, or task and being part of the same conversation (letting the same words activate relevant information in each partner). It is therefore a much more automatic and resource-cheap process than actually drawing inferences about the other’s knowledge. This mechanism takes into account the fact that the interlocutors are co-present on many different timescales (in culture, in multiple social projects, in a particular task, in a particular project within a task). The world, as its best model (Brooks, 1990), acts on both interlocutors alike.

What is still missing, again, is the distributed nature of the dialogical system: a mechanism for specialization in a task and bringing pooled, not only shared resources to bear on the dyad’s effectiveness. The pooled ground concept is thus based on the fact that different cultural and experiential history of the participants in an interaction will make the activated knowledge that guides behavior different for each interlocutor. This has often been viewed as a trouble, and possible cause of misunderstanding. However, this very same fact is the dyad’s strength, allowing for an optimal use of the potential resources. Thus, diversity is for good and for ill: for good because the idiosyncrasy of knowledge makes the knowledge base, upon which a dyad acts, much broader; for ill because it inevitably leads to misunderstandings and cases of miscommunication. It seems that much research has been devoted to the causes of misunderstandings treated as failures of communication, while in this light they can be seen as inevitable consequences of scouting for broader ground on which the interaction may build. Without misunderstandings, the discovery of relevant diversities would not be possible.

The concept of pooled ground has perhaps a stronger affinity to what Brown-Schmidt, one of the very few researchers who strives to go “beyond common and privileged ground” and toward task-immersed interactions, has called “potential” common ground: *“(…) that interlocutors would treat the common ground status of potential discourse referents as a gradient phenomenon sensitive to various sources of information in the discourse context*” (Brown-Schmidt, 2012, p. 65). The trick is to make this potentiality exert its influence without ever becoming common, leading to a truly distributed, and thus economical system, functioning according to the least collective effort principle.

## APPLICATION: PERSPECTIVE-TAKING IN DIALOG

To summarize, the synergetic notion of dialog, which views language as a system of constraints functionally controlling interactions, has a potential of clarifying the relationship between the two kinds of coordination previously recognized in dialog (Clark, 1996). The coordination on the linguistic level means establishing controls that are appropriate for the coordination on joint projects. The principle of least collaborative effort pertains to both levels: (i) enforcing the sparse (thrifty) use of constraints on an ongoing dynamics, and making both partners contribute to their construction and (ii) distributing the roles to make the dyad less redundant and more effectively using the resources, pooling them adequately to the situation.

Adding the collective level of situated interaction to the explanatory apparatus, with its qualitatively new resource in the form of the pooled ground, allows to see in a different light some of the recurrent problems in psycholinguistics. For the purpose of this paper, we have chosen to focus on perspective-taking in dialog. Perspective-taking is a case of a broader phenomenon in dialog research, namely audience design, and after a detailed analysis of the former, we also draw implications for this more general notion.

Factors that determine which perspective (allo- or egocentric) is taken in a given moment of an interaction have been a subject of intense debate over the last 15 years. According to Clark et al.’s (1983) theoretical proposition mentioned above, interlocutors should immediately restrict their interpretations according to the perspective of the interlocutor, narrowing it to the common ground. However, in the work of Keysar et al. (1998, 2000, 2003), this principle has been questioned by the results that show that addressees consider particular objects to be potential referents, even if these objects are not in common ground with the speaker (not visible to the speaker). When the commands from the speaker (e.g., “take a small candle”) were ambiguous and referred to a mutually visible object as well as to an object hidden from the speaker but visible to the addressee, participants in the addressee role often fixated on the hidden object first, indicating that they perceived it as a possible referent. The presence of a hidden semantic competitor made the time of interpretation longer compared to a situation without such a competitor. Sometimes, participants showed even more ‘grave’ egocentric mistakes by reaching for, or even grasping, the object in the privileged ground (Keysar et al., 2000).

These results were interpreted as evidence for a default egocentric perspective in communication. Keysar et al. (2000) proposed a model of perspective-taking in dialog, a perspective-adjustment model, in which interpretation is an egocentric process, with mechanisms of late adjustment to the speaker’s perspective activated only in cases of misunderstanding. Additional evidence for egocentric strategies in communication has been provided by research on cognitive costs of perspective-taking. Lin et al. (2010) showed that the ability to ignore the privately accessible part of a visual area in conversation correlates with executive resources such as working memory and inhibitory control. Other studies have confirmed that reasoning about others’ perspective indeed might not be automatic, even for adults (Apperly et al., 2007), which seemed to further support the egocentric model.

Despite their influence on interpretation theories, Keysar et al.’s (2000, 2003) studies were criticized on methodological grounds. As Hanna et al. (2003) noticed, objects in privileged ground which had to be ignored by participants were chosen in Keysar’s setups in such a manner that they were the best perceptual or semantic match for the descriptions (for example, they were the most typical referents). Consequently, participants had to resolve two conflicts: perspective discrepancy and lexical conflict. Lexical description pointed to the most typical referent, while shared perspective pointed to the less typical object visible for both participants. In Hanna et al. (2003), where lexical competition was under control, results showed that participants focused mostly on the shared objects, already from the beginning of the interpretation process. Nonetheless, they did look at the semantic competitors in privileged ground longer than at other irrelevant objects, so the perspective information was not the only type of information that determined behavior.

Accounting for these and other similar results which could not be readily encompassed within the Keysar’s model, Hanna et al. (2003), Hanna and Tanenhaus (2004) and Brown-Schmidt and Hanna (2011) proposed and refined a different model of perspective-taking in dialog, namely the constraint-based model. The model emphasizes the probabilistic and incremental nature of the interpretation process, where, from the beginning, different constraints (prosodic, syntactic, semantic, pragmatic, etc.) influence interpretation. The final interpretation depends on the strength of each constraint and on the competition among them. It may happen that despite the active perspective-taking (being in common ground) constraint, a stronger saliency constraint wins at the beginning of the interpretation process, focusing attention on the privileged but very salient object. Importantly, the constraint-based model allows for embracing relevant influences from different sources in the course of communication. Perhaps even those that were traditionally neglected and that stem from the joint nature of conversation.

This was shown in Duran and Dale’s (2014) recent study (see also Duran et al., 2011). The goal was to show that both egocentric and other-centric biases are simultaneously activated and compete for expression. The likelihood of eventually choosing one over the other depended, among other factors, on the information about the speaker’s capabilities. In their task, participants were required to interpret verbal instructions from a partner speaking from a specific spatial location with respect to the study participant, who directed them to select an object on a computer screen. Although participants in interaction were not physically co-present, the spatial referent was ostensibly visible to both the speaker and the addressee, albeit from different angles. Occasionally, instructions could be ambiguous with respect to which object (e.g., one on the left or the other on the right, depending on whose perspective was taken) should be selected.

Depending on additional information available on their partner (they were informed that the partner was either real or simulated), participants grounded interpretation either from their own visual perspective (i.e., egocentric stance) or from the visual perspective of their partner (i.e., other-centric stance). They did the latter more often if the speaker was known to be simulated, evidently preferring the egocentric stance if they knew they interacted with a live interlocutor who was able to (1) take their point of view if necessary and (2) ask a clarifying question in case of equivocation. Thus, the behavior of the participants was congruent with the least collaborative effort principle: putting less effort (egocentric perspective) when some effort was expected to be shared by a partner. In the case of a simulated partner, incapable of collaboration, other-centric responding was shown to be not only more frequent but also faster. Additionally, measuring the shape of response trajectories, the authors demonstrated that competition from an egocentric tendency was weaker in this condition.

Duran and Dale (2014) also showed, compatibly with the constraint-based model, that the data obtained were well accounted for by a dynamical model, in which the two perspectives are defined as attractors of individual dynamics. Attractors co-exist, and which one is chosen depends on their relative strength, which is influenced by the beliefs about the partner in interaction. What is crucial, though, is that the speeds of the participants’ reactions and the form of their behavior (the shape of trajectories for reaching the goal) were influenced by a mere presence of the non-chosen attractor. This illustrates what was mentioned earlier: information that potentially is relevant for the task and only potentially can enter common ground nevertheless exerts its influence on the ongoing interaction.

The above shows how the dialogical, joint nature of conversation brings in valid and important constraints that, together with the knowledge of common ground, co-determine the perspective taken on a concrete scene. However, most of the experimental work of Hanna and Tanenhaus (2004) and Brown-Schmidt and Hanna (2011) as well as the work of Duran and Dale (2014) pertain to rather limited situations, congruent with those traditionally studied in research on communication: agreeing on reference and resolving ambiguities, where the task-relevant objects are – by experimental situation design – presented in common ground. It is worth noticing, as some already have (Brown-Schmidt, 2012), that situations chosen for studies mostly involve interpretation of descriptions or imperatives, which require focusing on common ground and rarely, for example, the informational questions, which would require focusing on the privileged ground. Thus again, the tasks were chosen to study how people understand each other and not how they are able to form distributed functional systems. Yet it seems logical that if linguistic interactions are to broaden the capabilities of a dyad, it is precisely the private, or privileged, information that should be in focus. In fact, in one study by Brown-Schmidt (2009), participants were asked about an object in their private ground with an informational question. In this situation, they clearly focused more on the privileged ground target than on the common ground competitor, showing sensitivity to the speaker’s informational demands.

The power of an interacting system comes from its distributed nature. Using the concept of pooled ground helps understand how perspective taking may serve the global organization of a dyad. If we look at most real-life situations from a global perspective of an interactive dyad, pooling the ground for a dyadic system immersed in a task requires first scouting for information that potentially might be relevant for the task and volunteered or signalized in a collaborative interaction and then zooming on appropriate linguistic controls that coordinate this information. Keysar et al.’s (1998, 2000, 2003) results, as well as the slight initial egocentric bias found in almost all the above studies, might thus be taken not as evidence for egocentricity but as a preparation for being a valid partner in an interaction, able to contribute, or signal, idiosyncratic information or competence. Experimental setups where the participant sees that some information is blocked from the partner’s view lead to an increased responsibility for this very information in this participant (he is the only one who has access to it) and thus increases the tendency to focus on it.

We thus see an increasing flexibility in the models of perspective-taking: from Clark’s automatic initial adjusting to common ground (e.g., Clark and Carlson, 1981), or Keysar’s automatic, initial egocentricity (e.g., Keysar et al., 1998) to the constraint-based model of Hanna and Tanenhaus (2004) and Brown-Schmidt (2009), where perspective-taking depends on interaction of various factors (lexical, perspective of the partner, capabilities of the partner). In the next step (in Duran and Dale, 2014), the constraints are shown to be co-present and dynamically influence perspective-taking decisions. This emphasizes the joint, dialogical, nature of communication and the principle of the least collective effort. The synergetic model, underscoring both the jointness and the distributed nature of the conversing system, which requires pooling the participants’ resources, makes it possible to generalize the constraint-based model to other situations than ambiguity resolution or agreeing-on-reference, by letting various structures of the task determine the shape of the linguistic exchange and thus better predict conversational moves and the focus of attention. This, however, is possible only if we let the global level (the functional synergy) exert its influence, determining the distribution of complementary roles.

Rising to the level of interaction for explanatory variables has consequences for the phenomenon of audience design in general, of which perspective-taking is an example. The usual focus of the studies is on the ability of the speaker to adjust the utterance according to her beliefs about the knowledge or social status of the listeners (Clark and Krych, 2004; Horton and Gerrig, 2005). Addressees’ reactions are rich in cues about their conversational needs, which has been elegantly demonstrated by Kuhlen and Brennan’s (2013) work that led to questioning the validity of using confederates in some studies of interactive dialog. For example, in Brown and Dell (1987) experiment participants told a story to an allegedly naïve partner, who in fact was a confederate. In that case, participants were not eager to take the alleged addressees needs into account, which was interpreted as a proof of egocentrism. However, in the Lockridge and Brennan (2002) replication, when the confederate was replaced by an actual naïve partner who heard a story for the first time and who was allowed to give feedback to the speaker, participants showed sensitivity toward the addressee’s lack of knowledge already in the early stages of utterance production. This strong effect of the interlocutor presence suggests that parties in a dialog are actually very skillful in estimating the knowledge and conversational needs of a partner during dialog.

Focus on ‘doing together,’ however, leads one to ask a question whether, perhaps, participants are equally skillful in recognizing potentialities and not only needs of the others. Isaacs and Clark (1987), in their study on audience design, showed that recognition of the expertise level with respect to the task material is almost immediate, determining both experts’ and novices’ way of referring to objects. Perhaps the principle of least collaborative effort and the distributed nature of joint project realization, with the notion of pooled ground, can thus be useful also for generalizing principles of audience design: from offering information to be understood to designing contributions to get what is needed for interaction to go further. Such framework can be helpful in broadening the investigation of interaction to the contexts beyond the tasks that require zooming in on the same reference in common ground. In other contexts, audience design serves not only to supply information but also to seek information from a more knowledgeable partner: expressions are designed to get to the privileged ground but only as much as is needed to make our own next move.

## CONCLUSION

Pragmatic approaches see language as immersed in a variety of social projects. This perspective, taken in conjunction with dialogical and collective view on meaning-making, points to the fact that realization of a project often requires the coordination of distributed resources.

The notion that a global level of interaction may possess causal and functional properties is advocated by enactive approaches to cognition (Di Paolo et al., 2008) and, in the domain of linguistic functioning, by the model of dialog as interpersonal synergy (Fusaroli et al., 2014). At this level, with respect to collective goals, complementary roles for participants in a synergy are defined. Within such a framework, the use of language in interaction is thus responsible not only for creating and maintaining coherence and mutual understanding but also for distributing the roles in a task-dependent and complementary fashion. To describe the resource available to a dyad in this process, the notion of ‘pooled ground’ was proposed, which pertains to the level of interaction as a whole and comprises both the mutually known common ground and the elements of privileged grounds that may enter the common ground or may never do so, nevertheless having a causal role in constraining the dialogical system’s behavior.

Just as the alternative attractor in the Duran and Dale (2014) study that exerted influence on the shape of reaching trajectories, the privileged knowledge will have an influence on a speaker’s utterances (both the content and the way they are made), making them act slightly differently as constraints on the listener simply by virtue of being different physical controlling signals. This brings us back to the distinction between the physical and the semantic, which was made in note 3 at the beginning of this paper: in the framework in which language is understood as a constraint on an ongoing interaction, it is easier to see how the physicality of an utterance may become meaningful in a given situation.

The synergetic model leads to the reinterpretation of seemingly egocentric behaviors in perspective-taking as dyad-oriented; namely, they may stem from ‘scouting’ for useful task-relevant information. Similarly, audience design of utterances should be understood with respect to the joint project realized, and not as motivated solely by understanding each other. The emphasis on pooling and not equalizing the ground may show in a different light the problem of misunderstandings. They are a natural consequence of scouting for broader resources; their appearance is not only a signal that something should be repaired but, equally valuably, a signal of a potentially usable difference. They stem from constantly testing privileged information that can be volunteered or signalized in an interaction. The collective, distributed sense-making would thus not be possible without misunderstandings.

Balancing the synchrony/complementarity factors in a synergy leads to novel predictions about communicative behavior. It may, for example, be useful in determining the ‘degree of novelty’ that will be accepted in a conversation. In a situation of a strong need for group coherence, one might predict a heavier redundancy, i.e., staying within common ground (an emphasis on communion and the phatic aspect of an encounter) rather than risking miscommunication while scouting for maximal gain.

The theoretical and empirical focus in psycholinguistic studies exclusively on language, on linguistic exchanges and their ‘understanding,’ leads to underappreciation of a richly structured interaction constrained by many factors being already in place. Viewing linguistic interactions first as interactions on joint projects, with language as a source of constraints that structure them and divide labor, removes the explanatory burden of meaning-making, and understanding from language alone and poses it in the study of interaction in its context. With the pooled ground over both participants as resource, these interactions, as distributed collective structures, can be truly richer and more able than each of the participants alone.

## Conflict of Interest Statement

The authors declare that the research was conducted in the absence of any commercial or financial relationships that could be construed as a potential conflict of interest.

## Footnotes

1 Such a view of the role of language in situated cognition is congruent with Hutchins (1995) distributed cognition approach, where the focus is the ability of individuals to form collective functional organizations. In this approach, the collective, global level assumes a systemic property. Both local and global factors in cognition and action are investigated. When we refer to a ‘system’ in this text, we mean such an organization of individuals. Obviously, every such organization is situated in a particular environment that shapes it in different time scales. It is thus possible to conceive organisms-environment organization as a system as well. This is a matter of focus. In this paper, we chose to focus on human interaction – mostly dyadic but scalable to more participants – and treat environmental factors as constraints on this system.

2 “Functional control” is a term in motor control theory (from which the notion of ‘synergy’ has been adopted). Functional control is exerted through reducing the degrees of freedom of the parts of the system in a specific way, enabling a system to perform a coordinated movement, adequate to an ongoing activity (e.g., Bernstein, 1967).

3 This distinction might not be easy to make in some embodied accounts of cognition, where physical systems, due to their structure, shaped by natural selection, can also be seen as meaningful and intentionally committed to projects in the world (e.g., Merleau-Ponty, 1963). In such embodied view, physical interaction between the living system and the world, and among systems, can thus also be meaningful. Being aware of this, we preserve the distinction for the clarity of discourse and possible connection to research performed in more traditional approaches. See also the comments in Conclusions.

4 In the light of what follows, however, it is possible that if Tom knows that the hidden bottle better suits the purpose of Ann, he could reach for it (or direct his gaze toward it), even knowing that Ann refers to a commonly known bottle.

5 In this paper we make a strong assumption that collaboration is our species’ most prevalent mode. This does not preclude local competition and diversity – because in the slower time-scale they lead to more flexibility and better exploration of possibilities. Recently it seems increasingly popular to accept that the collective-collaborative level can be selected for as well (Christakis and Fowler, 2009; Smaldino, 2014).

6 Authors are grateful to the anonymous reviewer for pointing out this affinity. Indeed, there are more parallels between the view of language as social coordination advocated here and Garfinkel’s ethnomethodology. Perhaps most importantly, Garfinkel treats all utterances as indexical, therefore under-defined and always relying on the context of co-action. This under-definition is a key element in the framework that treats language as a system of replicable constraints on interactive events.

7 Here, by ‘mechanisms’ we mean processes that are sources of forces that make the coherence and complementarity possible. The trouble, however, is that in the case of such multisystem and multi-timescale phenomenon as language, those forces may be difficult to localize. On the one hand, it certainly is not enough to search for them only at the level of individual mind/brain; on the other hand, taking all the relevant systems and timescales into account might not be feasible in the process of theory construction. Here, we limit ourselves to those processes that produce structuring forces on the level of the individual and on the level of interaction, and limit the time scales to those of ongoing interaction and cultural evolution, bracketing out processes on different timescales while being aware of their presence. For a more detailed discussion of the multisystemic and multi-timescale nature of language, see Rączaszek-Leonardi (2003, 2010, 2014) or Enfield (2013). For how this influences the form of linguistic theory, see Rączaszek-Leonardi (2012)

8 Contrary to common ground, where a strict interpretation assumes that “B knows that A knows that B knows x,” in pooled ground this does not seem necessary. B does not have to know that A knows that she knows x. For example, B may speak/behave like an expert to A without knowing that she is one; however, she might also know this and therefore design her expressions accordingly.
